# 2,4,6-Trimethyl-3,5-bis[(phenylcarbono­thioyl)sulfanylmethyl]benzyl benzenecarbodithioate

**DOI:** 10.1107/S1600536810016028

**Published:** 2010-05-19

**Authors:** M. Kannan, V. Ramkumar, R. Dhamodharan

**Affiliations:** a Department of Chemistry, IIT Madras, Chennai, TamilNadu, India

## Abstract

In the title compound C_33_H_30_S_6_, the three pendant methyl­ene benzodithio­ate groups lie to one side of the central benzene ring in a *cis-cis-cis* ‘tripod’ arrangement. The dihedral angles between the central benzene ring and the three pendant rings are 72.54 (4), 89.68 (4) and 86.74 (4)°. In the crystal structure, one of the benzene rings is disordered over two orientations in a 0.559 (13):0.441 (13) ratio.

## Related literature

For applications of the title compound, see: Stenzel-Rosenbaum *et al.* (2001 ▶); Chong *et al.* (1999 ▶); Takolpuckdee *et al.* (2005 ▶). For a related structure, see: Li *et al.* (2002 ▶). 
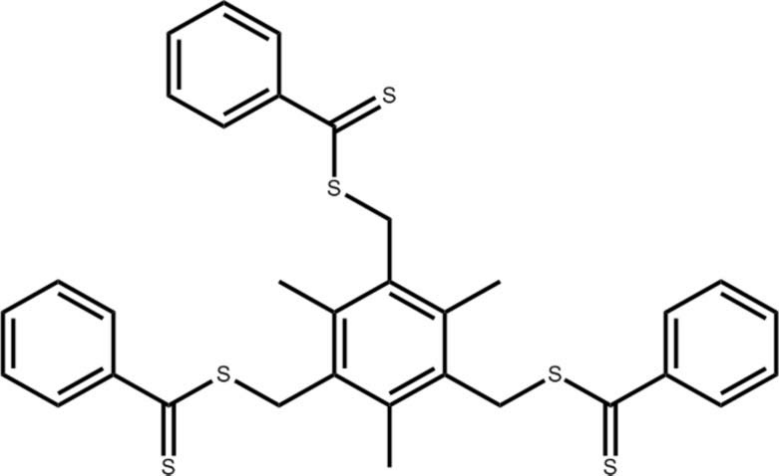


## Experimental

### 

#### Crystal data

C_33_H_30_S_6_
*M* *_r_* = 618.93
Monoclinic, 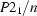

*a* = 9.5698 (3) Å
*b* = 21.7668 (10) Å
*c* = 15.3823 (8) Å
β = 94.819 (2)°
*V* = 3192.9 (2) Å^3^
*Z* = 4
Mo *K*α radiation
μ = 0.45 mm^−1^
*T* = 298 K
0.40 × 0.22 × 0.20 mm

#### Data collection

Bruker APEXII CCD diffractometer
Absorption correction: multi-scan (*SADABS*; Bruker, 1999 ▶) *T*_min_ = 0.841, *T*_max_ = 0.915
23638 measured reflections
7773 independent reflections
3544 reflections with *I* > 2σ(*I*)
*R*_int_ = 0.035

#### Refinement

*R*[*F* ^2^ > 2σ(*F* ^2^)] = 0.059
*wR*(*F* ^2^) = 0.188
*S* = 1.01
7773 reflections
363 parameters
1 restraint
H-atom parameters constrained
Δρ_max_ = 0.68 e Å^−3^
Δρ_min_ = −0.43 e Å^−3^

### 

Data collection: *APEX2* (Bruker, 2004 ▶); cell refinement: *APEX2* and *SAINT-plus* (Bruker, 2004 ▶); data reduction: *SAINT-Plus* and *XPREP* (Bruker, 2004 ▶); program(s) used to solve structure: *SHELXS97* (Sheldrick, 2008 ▶); program(s) used to refine structure: *SHELXL97* (Sheldrick, 2008 ▶); molecular graphics: *ORTEP-3* (Farrugia, 1997 ▶); software used to prepare material for publication: *SHELXL97*.

## Supplementary Material

Crystal structure: contains datablocks global, I. DOI: 10.1107/S1600536810016028/hb5411sup1.cif

Structure factors: contains datablocks I. DOI: 10.1107/S1600536810016028/hb5411Isup2.hkl

Additional supplementary materials:  crystallographic information; 3D view; checkCIF report

## Figures and Tables

**Fig. 1.**
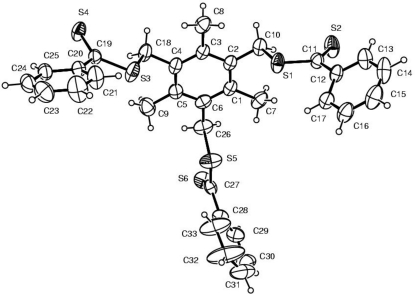
The molecular structure of (I) with atoms represented as 30% probability ellipsoids.

## supplementary crystallographic information

### Comment

The title compound C_33_H_30_S_6_ is a tri-functional dithioester derivative, which is used as a chain transfer agent (CTA) (Chong *et al.* 1999) in reversible addition fragmentation chain transfer (RAFT) polymerization. Being a tri-functional unit it can form the core of star polymer (Stenzel *et al.* 2001) when used as a CTA. Most of the reported mono functional CTAs are liquid and hence, very few single crystal XRD reports are available (Takolpuckdee *et al.* 2005). In the case of the multi-functional CTAs, depending upon the core it would be either solid or liquid. Since most of the synthesized multi-functional CTAs are characterized by other techniques, their single crystal XRD reports are not available. Here we report the title compound which is one such multi functional CTA, crystallised from hexane.

The title compound C_33_ H_30_ S_6_ adopts a *cis*,*cis*,*cis*- conformation where the three pendant arms (methylene benzodithioate) protrude on one side of the mean plane of the central benzene ring. Similar structures have been reported (Li *et al.*, 2002) where the three pendant arms (phenylthio groups) adopt *cis*,*trans*, *trans*- conformation. The replacement of phenylthio groups by benzodithioate groups flips the conformation from *cis*,*trans*, *trans* (`soft-shelled crawling turtle') to *cis*,*cis*,*cis*- conformation ('tripod stand').

The dihedral angle between the central benzene ring and the three methylene benzodithioate groups are 72.54 (4)°, 89.68 (4)° and 86.74 (4)°. The torsion angle of the three methylene benzodithioate group C2—C10—S1—C11, C4—C18—S3–C19 and C6—C26—S5—C27 are 147.1 (3),-174.8 (3) and -179.3 (3)°, respectively.

### Experimental

Phenyl magnesium bromide was prepared *in-situ* by adding bromobenzene (5 mmol) to activated Mg (5.5 mmol) in dry THF and the solution was refluxed for 1 h. To this reaction mixture carbon disulfide (5.5 mmol) was added over 10 min at 273 K. The mixture was allowed to warm to room temperature. Then 1,3,5-tris(bromomethyl)-2,4,6-trimethylbenzene (1 mmol) was added over 15 min. The reaction mixture was then placed in a constant temperature bath stirred at 323 K for 3 h and concentrated under reduced pressure. The resulting crude product was dissolved in ether, rinsed thrice with water, followed by brine solution and dried over anhydrous magnesium sulfate. The crude product was purified by column chromatography using 10% ethyl acetate in hexane as the eluent to obtain the pure title compound as a bright red solid. Recrystallization of the compound from hexane gave red blocks of (I).

### Refinement

All hydrogen atoms were fixed geometrically and allowed to ride on the parent carbon atoms, with aromatic C—H = 0.93 Å, methyl C—H = 0.96 Å and methylene C—H = 0.97 Å. The displacement parameters were set for phenyl and methylene H atoms at U_iso_(H) = 1.2U_eq_(C) and methyl H atoms at U_iso_(H) = 1.5U_eq_(C). C29 and C30 of one methylene benzodithiote arm is disordered over two sites in a ratio of 44° and 56°.

### Crystal data

**Table:**

C_33_H_30_S_6_	*F*(000) = 1296
*M**_r_* = 618.93	*D*_x_ = 1.288 Mg m^−^^3^
Monoclinic, *P*2_1_/*n*	Mo *K*α radiation, λ = 0.71073 Å
Hall symbol: -P 2yn	Cell parameters from 4990 reflections
*a* = 9.5698 (3) Å	θ = 2.7–22.8°
*b* = 21.7668 (10) Å	µ = 0.45 mm^−^^1^
*c* = 15.3823 (8) Å	*T* = 298 K
β = 94.819 (2)°	Block, red
*V* = 3192.9 (2) Å^3^	0.40 × 0.22 × 0.20 mm
*Z* = 4	

### Data collection

**Table:**

Bruker APEXII CCD diffractometer	7773 independent reflections
Radiation source: fine-focus sealed tube	3544 reflections with *I* > 2σ(*I*)
graphite	*R*_int_ = 0.035
phi and ω scans	θ_max_ = 28.3°, θ_min_ = 2.3°
Absorption correction: multi-scan (*SADABS*; Bruker, 1999)	*h* = −9→12
*T*_min_ = 0.841, *T*_max_ = 0.915	*k* = −29→29
23638 measured reflections	*l* = −20→20

### Refinement

**Table:**

Refinement on *F*^2^	Primary atom site location: structure-invariant direct methods
Least-squares matrix: full	Secondary atom site location: difference Fourier map
*R*[*F*^2^ > 2σ(*F*^2^)] = 0.059	Hydrogen site location: inferred from neighbouring sites
*wR*(*F*^2^) = 0.188	H-atom parameters constrained
*S* = 1.01	*w* = 1/[σ^2^(*F*_o_^2^) + (0.0749*P*)^2^ + 1.3743*P*] where *P* = (*F*_o_^2^ + 2*F*_c_^2^)/3
7773 reflections	(Δ/σ)_max_ < 0.001
363 parameters	Δρ_max_ = 0.68 e Å^−^^3^
1 restraint	Δρ_min_ = −0.43 e Å^−^^3^

### Special details

**Table:**

Geometry. All esds (except the esd in the dihedral angle between two l.s. planes)are estimated using the full covariance matrix. The cell esds are takeninto account individually in the estimation of esds in distances, anglesand torsion angles; correlations between esds in cell parameters are onlyused when they are defined by crystal symmetry. An approximate (isotropic)treatment of cell esds is used for estimating esds involving l.s. planes.
Refinement. Refinement of *F*^2^ against ALL reflections. The weighted *R*-factor wR andgoodness of fit *S* are based on *F*^2^, conventional *R*-factors *R* are basedon *F*, with *F* set to zero for negative *F*^2^. The threshold expression of*F*^2^ > σ(*F*^2^) is used only for calculating *R*-factors(gt) etc. and isnot relevant to the choice of reflections for refinement. *R*-factors basedon *F*^2^ are statistically about twice as large as those based on *F*, and *R*-factors based on ALL data will be even larger.

### Fractional atomic coordinates and isotropic or equivalent isotropic displacement parameters (Å^2^)

**Table:**

	*x*	*y*	*z*	*U*_iso_*/*U*_eq_	Occ. (<1)
C1	0.1544 (3)	0.05751 (14)	0.5508 (3)	0.0658 (9)	
C2	0.0778 (3)	0.07500 (14)	0.4741 (2)	0.0612 (9)	
C3	−0.0514 (3)	0.10408 (14)	0.4769 (2)	0.0623 (9)	
C4	−0.1075 (3)	0.11213 (14)	0.5571 (3)	0.0684 (10)	
C5	−0.0318 (4)	0.09496 (16)	0.6348 (3)	0.0717 (10)	
C6	0.0991 (4)	0.06642 (15)	0.6311 (3)	0.0712 (10)	
C7	0.2994 (4)	0.02961 (17)	0.5468 (3)	0.0886 (12)	
H7A	0.2905	−0.0117	0.5251	0.133*	
H7B	0.3473	0.0292	0.6042	0.133*	
H7C	0.3519	0.0537	0.5086	0.133*	
C8	−0.1286 (4)	0.12723 (19)	0.3935 (3)	0.0865 (12)	
H8A	−0.0620	0.1409	0.3543	0.130*	
H8B	−0.1883	0.1608	0.4065	0.130*	
H8C	−0.1844	0.0947	0.3666	0.130*	
C9	−0.0902 (5)	0.1067 (2)	0.7219 (3)	0.1069 (15)	
H9A	−0.1512	0.1418	0.7171	0.160*	
H9B	−0.0144	0.1144	0.7654	0.160*	
H9C	−0.1419	0.0714	0.7382	0.160*	
C10	0.1363 (4)	0.06230 (16)	0.3885 (3)	0.0755 (10)	
H10A	0.0602	0.0549	0.3440	0.091*	
H10B	0.1946	0.0258	0.3935	0.091*	
C11	0.3706 (3)	0.09594 (16)	0.3015 (2)	0.0672 (9)	
C12	0.4644 (3)	0.14406 (16)	0.2717 (3)	0.0691 (10)	
C13	0.5167 (5)	0.1390 (2)	0.1906 (3)	0.0962 (13)	
H13	0.4962	0.1044	0.1565	0.115*	
C14	0.5993 (5)	0.1852 (3)	0.1602 (4)	0.1174 (18)	
H14	0.6318	0.1821	0.1051	0.141*	
C15	0.6332 (5)	0.2353 (2)	0.2109 (5)	0.1130 (18)	
H15	0.6887	0.2663	0.1902	0.136*	
C16	0.5863 (4)	0.2402 (2)	0.2916 (4)	0.0965 (13)	
H16	0.6121	0.2739	0.3264	0.116*	
C17	0.5010 (4)	0.19564 (18)	0.3221 (3)	0.0792 (11)	
H17	0.4676	0.2000	0.3768	0.095*	
C18	−0.2524 (4)	0.13992 (16)	0.5593 (3)	0.0835 (12)	
H18A	−0.2991	0.1225	0.6071	0.100*	
H18B	−0.3084	0.1309	0.5052	0.100*	
C19	−0.4041 (3)	0.24648 (15)	0.5844 (2)	0.0612 (8)	
C20	−0.4158 (4)	0.31403 (16)	0.5938 (2)	0.0637 (9)	
C21	−0.3297 (5)	0.35393 (19)	0.5555 (4)	0.1077 (16)	
H21	−0.2596	0.3391	0.5228	0.129*	
C22	−0.3475 (6)	0.4169 (2)	0.5657 (4)	0.131 (2)	
H22	−0.2877	0.4438	0.5400	0.158*	
C23	−0.4486 (6)	0.4397 (2)	0.6115 (4)	0.1091 (15)	
H23	−0.4598	0.4819	0.6168	0.131*	
C24	−0.5334 (5)	0.4008 (2)	0.6498 (3)	0.0922 (13)	
H24	−0.6029	0.4164	0.6825	0.111*	
C25	−0.5189 (4)	0.33840 (18)	0.6414 (2)	0.0745 (10)	
H25	−0.5791	0.3122	0.6681	0.089*	
C26	0.1786 (5)	0.04527 (17)	0.7143 (3)	0.0897 (12)	
H26A	0.2269	0.0070	0.7046	0.108*	
H26B	0.1146	0.0386	0.7591	0.108*	
C27	0.3891 (4)	0.07623 (15)	0.8423 (2)	0.0657 (9)	
C28	0.5103 (4)	0.11464 (17)	0.8736 (2)	0.0734 (10)	
C29	0.541 (2)	0.1707 (9)	0.8418 (17)	0.106 (4)	0.444 (13)
H29	0.4751	0.1884	0.8014	0.127*	0.444 (13)
C30	0.659 (2)	0.2029 (9)	0.8639 (16)	0.126 (5)	0.444 (13)
H30	0.6787	0.2388	0.8344	0.152*	0.444 (13)
C29A	0.4891 (16)	0.1805 (7)	0.8748 (12)	0.106 (4)	0.559 (13)
H29A	0.4036	0.1981	0.8556	0.127*	0.559 (13)
C30A	0.6036 (18)	0.2161 (8)	0.9062 (11)	0.126 (5)	0.559 (13)
H30A	0.5950	0.2582	0.9144	0.152*	0.559 (13)
C31	0.7455 (8)	0.1828 (4)	0.9268 (4)	0.150 (3)	
H31	0.8059	0.2089	0.9596	0.180*	
C32	0.7429 (6)	0.1233 (3)	0.9417 (3)	0.1197 (18)	
H32	0.8207	0.1043	0.9704	0.144*	
C33	0.6275 (5)	0.0890 (2)	0.9155 (3)	0.0947 (13)	
H33	0.6288	0.0470	0.9264	0.114*	
S1	0.23941 (10)	0.12712 (4)	0.35723 (8)	0.0838 (3)	
S2	0.39012 (14)	0.02285 (5)	0.28250 (9)	0.1074 (4)	
S3	−0.23485 (10)	0.22221 (5)	0.57331 (10)	0.0997 (4)	
S4	−0.53785 (10)	0.19992 (5)	0.58348 (8)	0.0873 (4)	
S5	0.30387 (13)	0.10484 (5)	0.74795 (8)	0.0999 (4)	
S6	0.34802 (13)	0.01222 (5)	0.88751 (8)	0.0956 (4)	

### Atomic displacement parameters (Å^2^)

**Table:**

	*U*^11^	*U*^22^	*U*^33^	*U*^12^	*U*^13^	*U*^23^
C1	0.0529 (19)	0.0435 (17)	0.102 (3)	−0.0026 (15)	0.012 (2)	0.0022 (18)
C2	0.0537 (19)	0.0422 (16)	0.089 (3)	−0.0039 (14)	0.0129 (18)	−0.0020 (16)
C3	0.0524 (18)	0.0427 (16)	0.092 (3)	−0.0024 (14)	0.0092 (18)	−0.0004 (17)
C4	0.056 (2)	0.0477 (18)	0.104 (3)	−0.0036 (15)	0.018 (2)	−0.0068 (19)
C5	0.072 (2)	0.056 (2)	0.089 (3)	−0.0163 (18)	0.021 (2)	−0.0066 (19)
C6	0.068 (2)	0.0529 (19)	0.091 (3)	−0.0124 (17)	−0.002 (2)	0.0078 (19)
C7	0.063 (2)	0.063 (2)	0.138 (4)	0.0059 (18)	0.002 (2)	0.006 (2)
C8	0.074 (2)	0.076 (2)	0.108 (3)	0.010 (2)	0.004 (2)	0.003 (2)
C9	0.116 (4)	0.102 (3)	0.108 (4)	−0.026 (3)	0.037 (3)	−0.016 (3)
C10	0.073 (2)	0.0525 (19)	0.104 (3)	−0.0045 (17)	0.026 (2)	−0.0056 (19)
C11	0.062 (2)	0.060 (2)	0.081 (3)	0.0077 (17)	0.0129 (18)	−0.0047 (18)
C12	0.0562 (19)	0.063 (2)	0.090 (3)	0.0115 (17)	0.0190 (19)	0.0035 (19)
C13	0.100 (3)	0.086 (3)	0.108 (3)	0.009 (2)	0.042 (3)	−0.002 (3)
C14	0.114 (4)	0.114 (4)	0.134 (5)	0.018 (3)	0.071 (3)	0.025 (4)
C15	0.082 (3)	0.087 (3)	0.175 (6)	0.005 (3)	0.047 (3)	0.028 (4)
C16	0.072 (3)	0.081 (3)	0.140 (4)	−0.009 (2)	0.024 (3)	0.002 (3)
C17	0.065 (2)	0.078 (3)	0.096 (3)	−0.001 (2)	0.015 (2)	0.000 (2)
C18	0.059 (2)	0.059 (2)	0.136 (4)	−0.0014 (17)	0.028 (2)	−0.018 (2)
C19	0.0589 (19)	0.064 (2)	0.063 (2)	0.0018 (16)	0.0155 (15)	−0.0032 (16)
C20	0.066 (2)	0.062 (2)	0.065 (2)	0.0060 (17)	0.0104 (17)	−0.0004 (17)
C21	0.118 (4)	0.069 (3)	0.145 (4)	−0.009 (2)	0.064 (3)	−0.011 (3)
C22	0.161 (5)	0.070 (3)	0.173 (6)	−0.017 (3)	0.072 (4)	0.000 (3)
C23	0.141 (4)	0.069 (3)	0.117 (4)	0.016 (3)	0.015 (3)	−0.016 (3)
C24	0.103 (3)	0.089 (3)	0.085 (3)	0.034 (3)	0.009 (2)	−0.011 (2)
C25	0.076 (2)	0.080 (3)	0.068 (2)	0.019 (2)	0.0104 (18)	−0.0026 (19)
C26	0.100 (3)	0.060 (2)	0.107 (3)	−0.026 (2)	−0.006 (2)	0.014 (2)
C27	0.083 (2)	0.0506 (18)	0.066 (2)	0.0023 (17)	0.0209 (18)	−0.0040 (16)
C28	0.091 (3)	0.061 (2)	0.067 (2)	−0.008 (2)	0.001 (2)	0.0029 (18)
C29	0.087 (9)	0.062 (5)	0.164 (14)	−0.009 (6)	−0.016 (7)	0.005 (7)
C30	0.138 (12)	0.085 (7)	0.151 (14)	−0.043 (7)	−0.015 (8)	0.009 (7)
C29A	0.087 (9)	0.062 (5)	0.164 (14)	−0.009 (6)	−0.016 (7)	0.005 (7)
C30A	0.138 (12)	0.085 (7)	0.151 (14)	−0.043 (7)	−0.015 (8)	0.009 (7)
C31	0.156 (6)	0.179 (7)	0.108 (5)	−0.087 (6)	−0.029 (4)	0.010 (4)
C32	0.106 (4)	0.160 (6)	0.087 (4)	−0.018 (4)	−0.020 (3)	−0.005 (4)
C33	0.106 (3)	0.094 (3)	0.081 (3)	−0.002 (3)	−0.011 (3)	−0.002 (2)
S1	0.0767 (6)	0.0507 (5)	0.1303 (9)	0.0011 (4)	0.0448 (6)	−0.0054 (5)
S2	0.1203 (9)	0.0640 (6)	0.1464 (11)	0.0082 (6)	0.0606 (8)	−0.0146 (7)
S3	0.0552 (6)	0.0626 (6)	0.1851 (13)	−0.0040 (4)	0.0333 (6)	−0.0255 (7)
S4	0.0607 (6)	0.0743 (6)	0.1307 (10)	−0.0039 (5)	0.0304 (6)	0.0019 (6)
S5	0.1170 (9)	0.0645 (6)	0.1114 (9)	−0.0320 (6)	−0.0304 (7)	0.0302 (6)
S6	0.1257 (9)	0.0739 (7)	0.0882 (8)	−0.0192 (6)	0.0141 (7)	0.0241 (6)

### Geometric parameters (Å, °)

**Table:**

C1—C2	1.389 (5)	C18—H18A	0.9700
C1—C6	1.398 (5)	C18—H18B	0.9700
C1—C7	1.520 (5)	C19—C20	1.483 (5)
C2—C3	1.393 (4)	C19—S4	1.632 (3)
C2—C10	1.499 (5)	C19—S3	1.726 (3)
C3—C4	1.398 (5)	C20—C21	1.364 (5)
C3—C8	1.512 (5)	C20—C25	1.383 (5)
C4—C5	1.396 (5)	C21—C22	1.392 (6)
C4—C18	1.516 (5)	C21—H21	0.9300
C5—C6	1.403 (5)	C22—C23	1.339 (7)
C5—C9	1.515 (5)	C22—H22	0.9300
C6—C26	1.504 (5)	C23—C24	1.342 (6)
C7—H7A	0.9600	C23—H23	0.9300
C7—H7B	0.9600	C24—C25	1.373 (6)
C7—H7C	0.9600	C24—H24	0.9300
C8—H8A	0.9600	C25—H25	0.9300
C8—H8B	0.9600	C26—S5	1.812 (4)
C8—H8C	0.9600	C26—H26A	0.9700
C9—H9A	0.9600	C26—H26B	0.9700
C9—H9B	0.9600	C27—C28	1.477 (5)
C9—H9C	0.9600	C27—S6	1.620 (3)
C10—S1	1.810 (3)	C27—S5	1.721 (4)
C10—H10A	0.9700	C28—C29	1.36 (2)
C10—H10B	0.9700	C28—C33	1.365 (5)
C11—C12	1.478 (5)	C28—C29A	1.447 (16)
C11—S2	1.631 (3)	C29—C30	1.35 (3)
C11—S1	1.718 (3)	C29—H29	0.9300
C12—C13	1.386 (5)	C30—C31	1.29 (2)
C12—C17	1.392 (5)	C30—H30	0.9300
C13—C14	1.385 (6)	C29A—C30A	1.39 (2)
C13—H13	0.9300	C29A—H29A	0.9300
C14—C15	1.364 (7)	C30A—C31	1.55 (2)
C14—H14	0.9300	C30A—H30A	0.9300
C15—C16	1.359 (7)	C31—C32	1.316 (8)
C15—H15	0.9300	C31—H31	0.9300
C16—C17	1.376 (5)	C32—C33	1.365 (7)
C16—H16	0.9300	C32—H32	0.9300
C17—H17	0.9300	C33—H33	0.9300
C18—S3	1.810 (4)		
			
C2—C1—C6	120.0 (3)	C4—C18—H18B	109.9
C2—C1—C7	119.6 (4)	S3—C18—H18B	109.9
C6—C1—C7	120.4 (4)	H18A—C18—H18B	108.3
C1—C2—C3	120.3 (3)	C20—C19—S4	123.4 (2)
C1—C2—C10	119.1 (3)	C20—C19—S3	113.1 (2)
C3—C2—C10	120.6 (3)	S4—C19—S3	123.4 (2)
C2—C3—C4	119.5 (3)	C21—C20—C25	117.9 (3)
C2—C3—C8	119.8 (3)	C21—C20—C19	122.4 (3)
C4—C3—C8	120.7 (3)	C25—C20—C19	119.7 (3)
C5—C4—C3	120.8 (3)	C20—C21—C22	119.6 (4)
C5—C4—C18	119.9 (4)	C20—C21—H21	120.2
C3—C4—C18	119.3 (4)	C22—C21—H21	120.2
C4—C5—C6	119.0 (3)	C23—C22—C21	121.6 (5)
C4—C5—C9	120.5 (4)	C23—C22—H22	119.2
C6—C5—C9	120.5 (4)	C21—C22—H22	119.2
C1—C6—C5	120.2 (3)	C22—C23—C24	119.2 (5)
C1—C6—C26	120.5 (4)	C22—C23—H23	120.4
C5—C6—C26	119.3 (4)	C24—C23—H23	120.4
C1—C7—H7A	109.5	C23—C24—C25	120.8 (4)
C1—C7—H7B	109.5	C23—C24—H24	119.6
H7A—C7—H7B	109.5	C25—C24—H24	119.6
C1—C7—H7C	109.5	C24—C25—C20	120.8 (4)
H7A—C7—H7C	109.5	C24—C25—H25	119.6
H7B—C7—H7C	109.5	C20—C25—H25	119.6
C3—C8—H8A	109.5	C6—C26—S5	107.4 (2)
C3—C8—H8B	109.5	C6—C26—H26A	110.2
H8A—C8—H8B	109.5	S5—C26—H26A	110.2
C3—C8—H8C	109.5	C6—C26—H26B	110.2
H8A—C8—H8C	109.5	S5—C26—H26B	110.2
H8B—C8—H8C	109.5	H26A—C26—H26B	108.5
C5—C9—H9A	109.5	C28—C27—S6	124.0 (3)
C5—C9—H9B	109.5	C28—C27—S5	112.0 (2)
H9A—C9—H9B	109.5	S6—C27—S5	123.9 (2)
C5—C9—H9C	109.5	C29—C28—C33	110.1 (9)
H9A—C9—H9C	109.5	C29—C28—C29A	31.6 (8)
H9B—C9—H9C	109.5	C33—C28—C29A	120.6 (7)
C2—C10—S1	110.0 (2)	C29—C28—C27	125.4 (10)
C2—C10—H10A	109.7	C33—C28—C27	120.9 (4)
S1—C10—H10A	109.7	C29A—C28—C27	117.2 (7)
C2—C10—H10B	109.7	C30—C29—C28	125 (2)
S1—C10—H10B	109.7	C30—C29—H29	117.4
H10A—C10—H10B	108.2	C28—C29—H29	117.4
C12—C11—S2	123.6 (3)	C31—C30—C29	118.7 (19)
C12—C11—S1	111.3 (2)	C31—C30—H30	120.6
S2—C11—S1	125.1 (2)	C29—C30—H30	120.6
C13—C12—C17	118.1 (4)	C30A—C29A—C28	116.6 (13)
C13—C12—C11	119.9 (4)	C30A—C29A—H29A	121.7
C17—C12—C11	122.0 (3)	C28—C29A—H29A	121.7
C14—C13—C12	120.4 (5)	C29A—C30A—C31	117.4 (12)
C14—C13—H13	119.8	C29A—C30A—H30A	121.3
C12—C13—H13	119.8	C31—C30A—H30A	121.3
C15—C14—C13	120.2 (5)	C30—C31—C32	116.3 (10)
C15—C14—H14	119.9	C30—C31—C30A	36.5 (9)
C13—C14—H14	119.9	C32—C31—C30A	117.8 (7)
C16—C15—C14	120.3 (5)	C30—C31—H31	121.8
C16—C15—H15	119.9	C32—C31—H31	121.8
C14—C15—H15	119.9	C30A—C31—H31	108.6
C15—C16—C17	120.5 (5)	C31—C32—C33	120.9 (6)
C15—C16—H16	119.7	C31—C32—H32	119.6
C17—C16—H16	119.7	C33—C32—H32	119.6
C16—C17—C12	120.5 (4)	C32—C33—C28	121.9 (5)
C16—C17—H17	119.8	C32—C33—H33	119.1
C12—C17—H17	119.8	C28—C33—H33	119.1
C4—C18—S3	108.7 (2)	C11—S1—C10	105.19 (17)
C4—C18—H18A	109.9	C19—S3—C18	103.65 (16)
S3—C18—H18A	109.9	C27—S5—C26	103.75 (18)
			
C6—C1—C2—C3	3.0 (5)	C25—C20—C21—C22	0.3 (7)
C7—C1—C2—C3	−176.0 (3)	C19—C20—C21—C22	179.0 (4)
C6—C1—C2—C10	−177.1 (3)	C20—C21—C22—C23	−0.8 (9)
C7—C1—C2—C10	3.8 (4)	C21—C22—C23—C24	1.1 (9)
C1—C2—C3—C4	−4.0 (5)	C22—C23—C24—C25	−0.9 (8)
C10—C2—C3—C4	176.2 (3)	C23—C24—C25—C20	0.5 (7)
C1—C2—C3—C8	175.3 (3)	C21—C20—C25—C24	−0.1 (6)
C10—C2—C3—C8	−4.5 (5)	C19—C20—C25—C24	−178.9 (3)
C2—C3—C4—C5	4.1 (5)	C1—C6—C26—S5	83.7 (4)
C8—C3—C4—C5	−175.2 (3)	C5—C6—C26—S5	−96.6 (4)
C2—C3—C4—C18	−175.9 (3)	S6—C27—C28—C29	−174.4 (10)
C8—C3—C4—C18	4.8 (5)	S5—C27—C28—C29	9.7 (11)
C3—C4—C5—C6	−3.3 (5)	S6—C27—C28—C33	29.1 (5)
C18—C4—C5—C6	176.7 (3)	S5—C27—C28—C33	−146.9 (3)
C3—C4—C5—C9	177.0 (3)	S6—C27—C28—C29A	−138.4 (8)
C18—C4—C5—C9	−3.0 (5)	S5—C27—C28—C29A	45.7 (9)
C2—C1—C6—C5	−2.2 (5)	C33—C28—C29—C30	−14 (2)
C7—C1—C6—C5	176.8 (3)	C29A—C28—C29—C30	102 (4)
C2—C1—C6—C26	177.5 (3)	C27—C28—C29—C30	−172.5 (13)
C7—C1—C6—C26	−3.5 (5)	C28—C29—C30—C31	−8(3)
C4—C5—C6—C1	2.4 (5)	C29—C28—C29A—C30A	−67 (3)
C9—C5—C6—C1	−177.9 (3)	C33—C28—C29A—C30A	11.2 (16)
C4—C5—C6—C26	−177.3 (3)	C27—C28—C29A—C30A	178.8 (9)
C9—C5—C6—C26	2.4 (5)	C28—C29A—C30A—C31	6.7 (18)
C1—C2—C10—S1	−90.9 (3)	C29—C30—C31—C32	26 (2)
C3—C2—C10—S1	89.0 (3)	C29—C30—C31—C30A	−76 (2)
S2—C11—C12—C13	37.9 (5)	C29A—C30A—C31—C30	75 (2)
S1—C11—C12—C13	−141.6 (3)	C29A—C30A—C31—C32	−22.0 (16)
S2—C11—C12—C17	−143.2 (3)	C30—C31—C32—C33	−22.3 (15)
S1—C11—C12—C17	37.3 (4)	C30A—C31—C32—C33	18.9 (12)
C17—C12—C13—C14	−2.0 (6)	C31—C32—C33—C28	−0.5 (9)
C11—C12—C13—C14	176.9 (4)	C29—C28—C33—C32	17.4 (12)
C12—C13—C14—C15	1.9 (7)	C29A—C28—C33—C32	−15.8 (10)
C13—C14—C15—C16	0.1 (8)	C27—C28—C33—C32	177.2 (4)
C14—C15—C16—C17	−1.8 (7)	C12—C11—S1—C10	−179.9 (3)
C15—C16—C17—C12	1.7 (6)	S2—C11—S1—C10	0.6 (3)
C13—C12—C17—C16	0.2 (6)	C2—C10—S1—C11	147.4 (3)
C11—C12—C17—C16	−178.6 (3)	C20—C19—S3—C18	−178.0 (3)
C5—C4—C18—S3	87.6 (4)	S4—C19—S3—C18	1.4 (3)
C3—C4—C18—S3	−92.3 (4)	C4—C18—S3—C19	−174.8 (3)
S4—C19—C20—C21	−148.1 (4)	C28—C27—S5—C26	171.9 (3)
S3—C19—C20—C21	31.2 (5)	S6—C27—S5—C26	−4.0 (3)
S4—C19—C20—C25	30.6 (5)	C6—C26—S5—C27	−179.7 (3)
S3—C19—C20—C25	−150.0 (3)		
